# Generalized granuloma annulare successfully treated with deucravacitinib (Sotyktu)

**DOI:** 10.1016/j.jdcr.2025.02.034

**Published:** 2025-03-14

**Authors:** Tatiana Lapa, Maksym Breslavets

**Affiliations:** a Department of Dermatology, University of Toronto, Toronto, Canada; b Centre for Medical and Surgical Dermatology, Pickering, Ontario, Canada

**Keywords:** deucravacitinib, generalized granuloma annulare (GA), Sotyktu

## Introduction

Granuloma annulare (GA) is an inflammatory granulomatous skin condition affecting primarily females in the first 3-5 decades of life. GA may present as localized or generalized/disseminated forms, which can be further categorized based on lesion depth as patch, perforating, or subcutaneous variants.^1^ While localized GA is often self-resolving, generalized GA tends to be recalcitrant to treatments. Management of generalized GA is challenging due to a lack of high-level evidence-based treatment options. The therapeutic approach typically begins with topical corticosteroids and calcineurin inhibitors, progressing to intralesional corticosteroids, followed by systemic treatments including methotrexate, sulfasalazine, cyclosporine, dapsone, antimalarials (eg, hydroxychloroquine), pentoxifylline, oral retinoids, chlorambucil, nicotinamide, fumaric acid esters, antithrombotic agents (eg, defibrotide), ROM therapy (a combination of rifampin, ofloxacin, and minocycline), surgical excision, phototherapy, and advanced systemic therapies for recalcitrant cases.^1^, ^2^, ^3^, ^4^, ^5^

Recent studies have explored small-molecule drugs, such as PDE4 inhibitor (apremilast) as well as novel biologic agents, including IL-4/13 inhibitors (dupilumab), IL-12/23 (Ustekinumab), IL-23 inhibitors (tildrakizumab), tumor necrosis factor-alpha inhibitors (adalimumab, etanercept, infliximab), anti-CD11a (efalizumab), and Janus kinase (JAK) inhibitors (baricitinib, tofacitinib, abrocitinib, upadacitinib), for resistant GA.^1^^,^^6^^,^^7^ We describe a case of generalized GA refractory to topical and oral corticosteroids, narrowband UVB phototherapy, and oral roflumilast, who achieved significant improvement and near-complete resolution of lesions with deucravacitinib (Sotyktu), a novel TYK 2 inhibitor.

## Case report

A 63-year-old male with no known past medical history, including diabetes or dyslipidemia, presented with a 1-year history of generalized GA and was referred to the clinic for ongoing management. Over the past year, he experienced multiple mildly itchy lesions on his trunk and upper extremities. Minimal relief was achieved with topical corticosteroids, a combination of corticosteroids with vitamin D analogues (Enstilar foam), and a course of systemic corticosteroids (oral prednisone, starting at 40 mg daily for 1 week, followed by a gradual taper).

**Fig 1:**
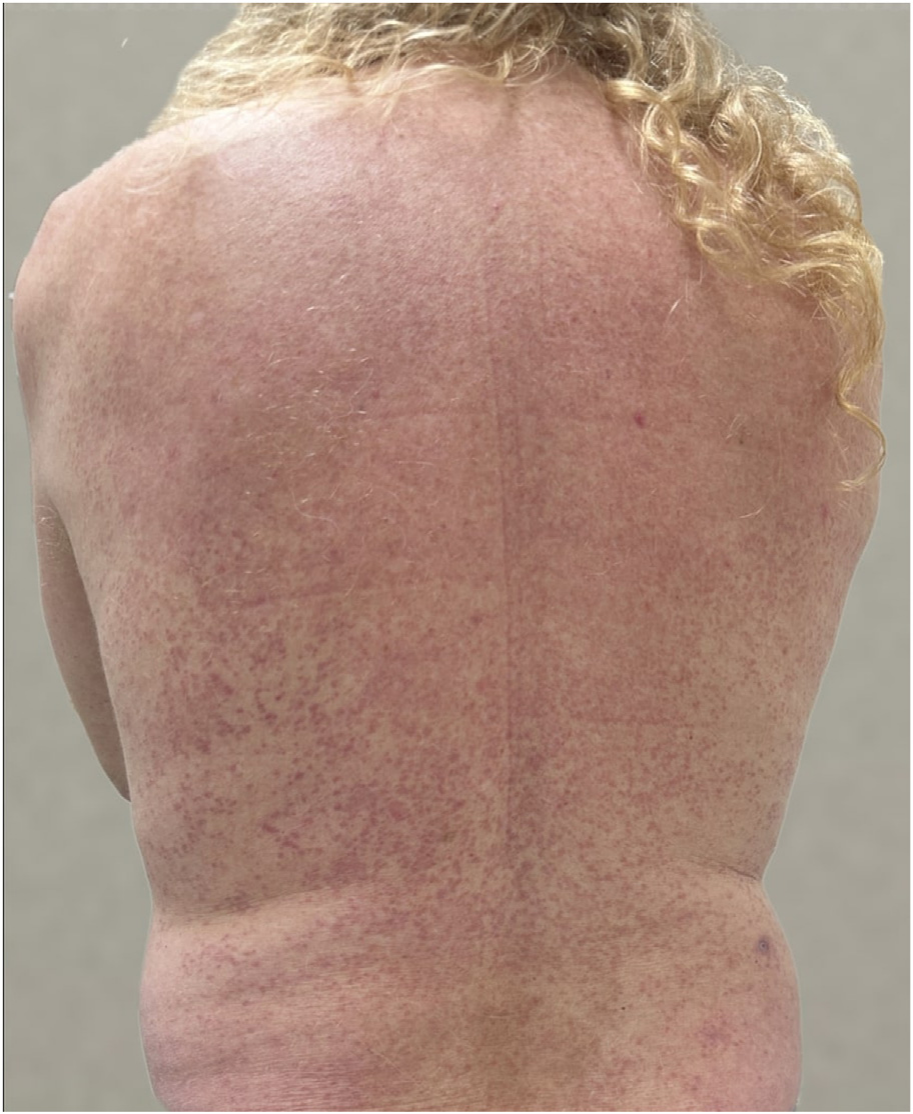
Generalized granuloma annulare before treatment (*back*).

**Fig 2:**
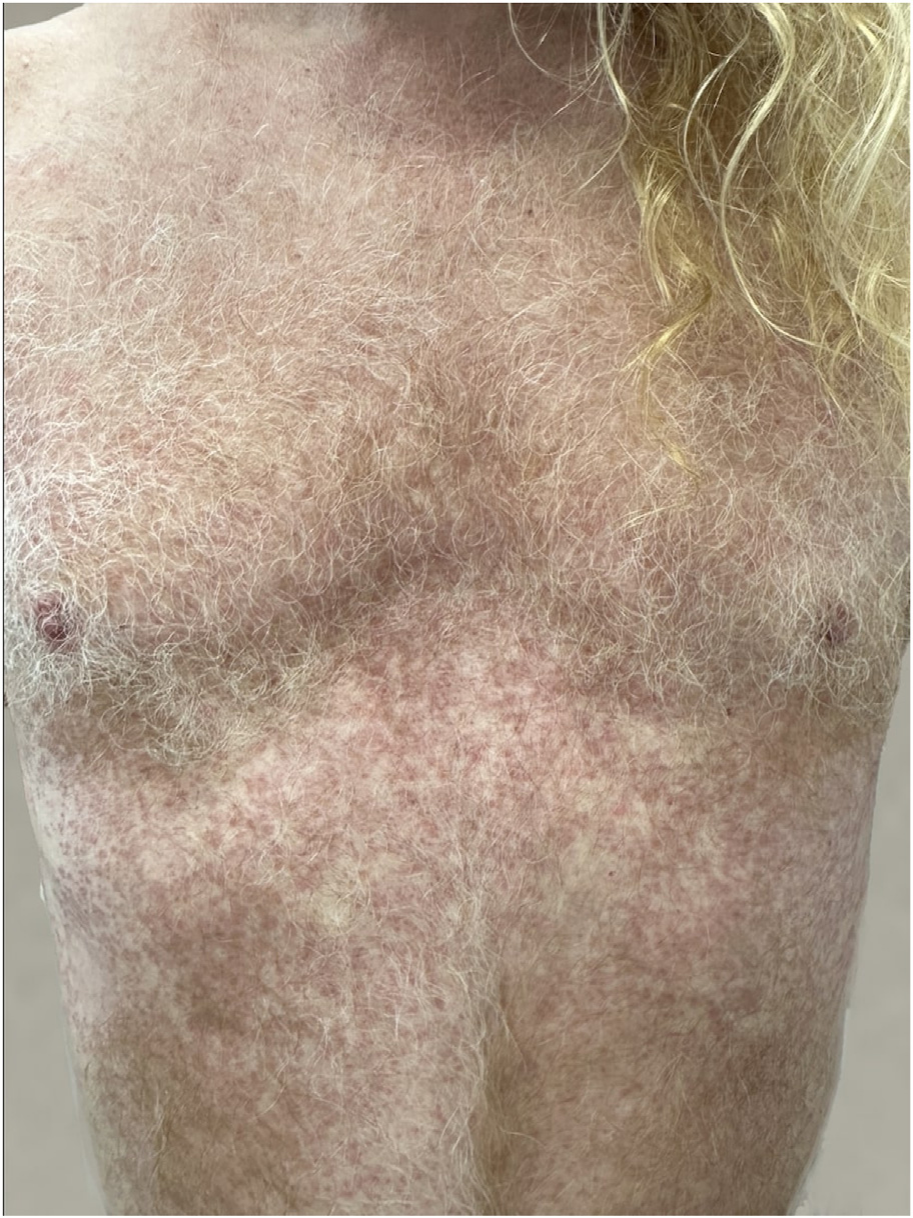
Generalized granuloma annulare before treatment (*front*).

On examination, he presented with multiple papules coalescing into annular plaques symmetrically distributed on the trunk and, to a lesser extent, on his left arm (Figs 1 and 2). A previous punch biopsy had findings compatible with GA. A repeat punch biopsy from the right flank revealed normal epithelium, focal mucin deposition between collagen bundles, surrounding ill-defined palisading granulomas, and a perivascular lymphocytic infiltrate in the dermis, confirming the diagnosis of generalized GA.

**Fig 3:**
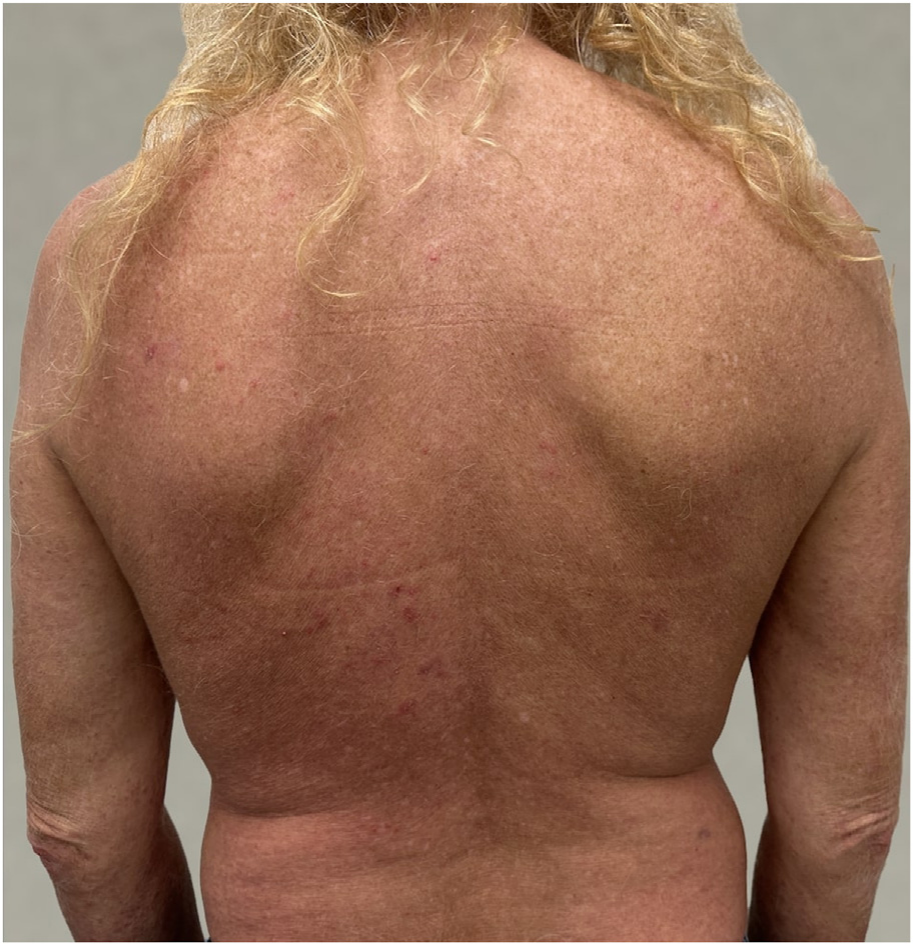
After a 3-month course of deucravacitinib (Sotyktu) (*back*).

**Fig 4:**
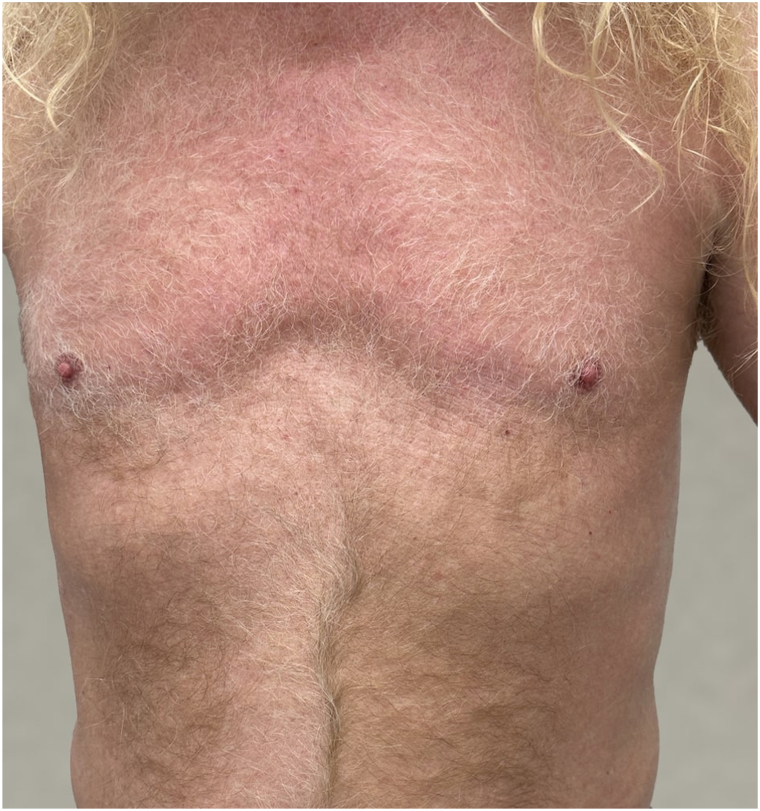
After a 3-m course of deucravacitinib (Sotyktu) (*front*).

The patient was started on roflumilast 500 mcg orally once daily in addition to prior topical therapies but showed limited improvement. A month later, narrowband UVB phototherapy was initiated, and a total of 42 sessions were completed, also yielding minimal effect. After 3 months of therapy, roflumilast was discontinued, and deucravacitinib (Sotyktu) 6 mg orally once daily was introduced, while narrowband UVB therapy continued. Before initiating deucravacitinib, a preimmunosuppressive workup, including lipid profile and a TB skin test, was completed. Within 3 months, the patient experienced significant improvement, with almost complete resolution of lesions on the trunk and left arm (Figs 3 and 4).

He continues to take deucravacitinib 6 mg daily, and the positive clinical response has been maintained at his 1-2-month follow-up. Ongoing blood work, including lipid profile monitoring, is conducted in accordance with treatment monitoring recommendations. Further follow-up is ongoing to monitor the long-term efficacy and safety of this treatment.

## Discussion

Disseminated or generalized GA is an uncommon form of the disease, typically presenting later in life and demonstrating a poorer response to conventional therapies.

The etiology and pathogenesis of GA remain unclear with potential triggers, including trauma, insect bites, tuberculin skin testing, vaccination, ultraviolet exposure, and infections (such as Borrelia, Chlamydia, and viruses including Epstein-Barr virus, HIV, varicella-zoster virus, and COVID-19).^1^ One prevailing hypothesis suggests a delayed-type hypersensitivity reaction to an unidentified antigen, mediated by a Th1 response, with elevated IL-2 receptor-positive lymphocytes, interferon-gamma producing lymphocytes, and increased tumor necrosis factor.^1^^,^^8^

Recent research has shown that GA lesions upregulate both Th1 and Th2 pathways, challenging earlier beliefs that primarily implicated the Th1 pathway.^9^ Min et al reported increased expression of Th1 cytokines (tumor necrosis factor-α, IL-1β, IFN-γ, and IL-12/23p40) alongside Th2 markers (IL-4 and IL-31), with IL-4 exhibiting a remarkable 15,600-fold increase in lesional skin.^9^

In addition to Th1 and Th2 involvement, Th17 and Th22 pathways and the Janus kinase-signal transducer and activator of transcription pathway have also been shown to be upregulated. A study by Wang et al in 2021 similarly reported activation of the Th1 and Janus kinase-signal transducer and activator of transcription pathways in GA. They identified elevated mRNA levels of key Janus kinase-signal transducer and activator of transcription mediators, such as IFN-γ and oncostatin M, and noted the upregulation of both “M1” and “M2” macrophage polarization.^4^ This dual macrophage response suggests a biphasic disease mechanism, with initial collagen degradation driven by M1 macrophages, followed by tissue remodeling and mucin deposition facilitated by M2 macrophages. This dual Th1/Th2 inflammatory profile, combined with Janus kinase-signal transducer and activator of transcription pathway involvement, highlights the significant immune dysregulation at play in GA.

The emerging understanding of these inflammatory pathways underscores the need for further research into targeted treatments. Evidence of significant JAK and Th2 pathway activation opens avenues for exploring JAK inhibitors and Th2-targeted therapies as promising options for treating recalcitrant GA.

Deucravacitinib (Sotyktu), an oral medication, allosterically inhibits tyrosine kinase 2, an enzyme that mediates signaling for key cytokines such as IL-23 and type I interferons, which are implicated in inflammatory diseases like psoriasis.^10^ Unlike traditional JAK inhibitors, which bind to the conserved catalytic domains of JAK1, JAK2, and JAK3, Deucravacitinib selectively targets tyrosine kinase 2’s regulatory domain. This selectivity results in substantial tyrosine kinase 2 inhibition with minimal impact on JAK1, JAK2, and JAK3. Clinical trials support deucravacitinib’s efficacy and favorable safety profile in moderate-to-severe plaque psoriasis, highlighting its potential as an off-label treatment for other dermatological conditions.

To our knowledge, this is the first reported case of successful deucravacitinib treatment for GA. Further clinical studies are necessary to assess the mechanism of action, efficacy, and long-term safety of deucravacitinib in treating GA.

## Conflicts of interest

None disclosed.
